# Angiotensin type 2 receptor antagonism as a new target to manage gout

**DOI:** 10.1007/s10787-022-01076-x

**Published:** 2022-09-29

**Authors:** Thiago Neves Vieira, André L. Lopes Saraiva, Rafaela Mano Guimarães, João Paulo Mesquita Luiz, Larissa Garcia Pinto, Veridiana de Melo Rodrigues Ávila, Luiz Ricardo Goulart, Jair Pereira Cunha-Junior, Peter Anthony McNaughton, Thiago Mattar Cunha, Juliano Ferreira, Cassia Regina Silva

**Affiliations:** 1 Graduate Program in Genetics and Biochemistry, Institute of Biotechnology, Federal University of Uberlândia, 38408-100 Uberlândia (MG), Brazil; 2 Center for Research in Inflammatory Diseases (CRID), Department of Pharmacology, Ribeirão Preto Medical School, University of São Paulo, Ribeirão Preto, SP, Brazil; 3 Wolfson Centre for Age-Related Diseases. King’s College London, Guy’s Campus, London SE1 1UL, United Kingdom; 4 Department of Immunology, Institute of Sciences Biomedical Sciences, Federal University of Uberlândia, 38405-318 Uberlândia (MG), Brazil; 5 Graduated Program in Pharmacology, Pharmacology Department, Federal University of Santa Catarina (UFSC), 88049-900 Florianopolis (SC), Brazil

**Keywords:** arthritis, monosodium urate crystals, pain, inflammation, IL-1β

## Abstract

**Background:**

There is a growing search for therapeutic targets in the treatment of gout. The present study aimed to evaluate the analgesic and anti-inflammatory potential of angiotensin type 2 receptor (AT_2_R) antagonism in an acute gout attack mouse model.

**Methods:**

Male wild type (WT) C57BL/6 mice either with the AT_2_R antagonist, PD123319 (10 pmol/joint), or with vehicle injections, or AT_2_R KO mice, received intra-articular (IA) injection of monosodium urate (MSU) crystals (100 μg/joint), that induce the acute gout attack, and were tested for mechanical allodynia, thermal hyperalgesia, spontaneous nociception and ankle edema development at several times after the injections. To test an involvement of AT_2_R in joint pain, mice received an IA administration of angiotensin II (0.05–5 nmol/joint) with or without PD123319, and were also evaluated for pain and edema development. Ankle joint tissue samples from mice undergoing the above treatments were assessed for myeloperoxidase activity, IL-1β release, mRNA expression analyses and nitrite/nitrate levels, 4 h after injections.

**Results:**

AT_2_R antagonism has robust antinociceptive effects on mechanical allodynia (44% reduction) and spontaneous nociception (56%), as well as anti-inflammatory effects preventing edema formation (45%), reducing myeloperoxidase activity (54%) and IL-1β levels (32%). Additionally, Agtr2^tm1a^ mutant mice have largely reduced painful signs of gout. Angiotensin II administration causes pain and inflammation, which was prevented by AT_2_R antagonism, as observed in mechanical allodynia 4 h (100%), spontaneous nociception (46%), cold nociceptive response (54%), edema formation (83%), myeloperoxidase activity (48%), and IL-1β levels (89%). PD123319 treatment also reduces NO concentrations (74%) and AT_2_R mRNA levels in comparison with MSU untreated mice.

**Conclusion:**

Our findings show that AT_2_R activation contributes to acute pain in experimental mouse models of gout. Therefore, the antagonism of AT_2_R may be a potential therapeutic option to manage gout arthritis.

## 1 Introduction

Gouty arthritis is characterized by hyperuricemia (serum urate levels ≥ 7 mg/L) that leads to the formation and deposition of monosodium urate (MSU) crystals in the joints, resulting in disabling pain. Gout is the most common cause of inflammatory arthritis worldwide (Dalbeth et al. 2018, 2021). However, for the large and growing number of individuals with gout, current therapeutic options remain limited and are largely contraindicated, mainly because of the concomitant presence of comorbidities that these individuals exhibit which reduce therapeutic efficacy, increase toxicity and make them prone to adverse effects of drug-drug interactions (Schlesinger 2017; Elfishawi et al. 2018).

Hypertension is among the most frequent comorbidities associated with gout. Some of the drugs used to treat hypertension, such as angiotensin converting enzyme inhibitors (ACEi) have been shown to increase the risk of developing an acute gout attack (Choi et al. 2012; Zhu et al. 2012; Elfishawi et al. 2018). It is well known that inhibition of angiotensin converting enzyme (ACE) can result in the upregulation of bradykinin and of the renin-angiotensin systems. Our group have demonstrated that the kinin system is only partially involved in an acute gout attack, including those precipitated by the use of ACEi (Silva et al. 2016). However, there are no studies to date evaluating the possible involvement of renin-angiotensin system in gout. We investigate here whether a dysregulation of the renin-angiotensin system, and in particular an action at the AT_2_R, may also be responsible for the pain and inflammation observed in gout.

The angiotensin system has two major G protein-coupled receptor subtypes, the angiotensin II type 1 receptor (AT_1_R), that plays an important role in the regulation of blood pressure, and the angiotensin II type 2 receptor (AT_2_R), that has recently been shown to play an important role in pain (Vargas et al. 2022). The AT_2_R is expressed in different cell types present in the articular environment, such as endothelial cells, synoviocytes, peripheral sensory neurons and peripheral macrophages (Pueyo and Michel 1997; Terenzi et al. 2017; Shepherd et al. 2018a). Recent findings demonstrate an involvement of the angiotensin system targeting AT_2_R in pain sensitization and, that AT_2_R antagonism has antinociceptive effects in animal models of neuropathic, inflammatory and bone cancer pain (Smith et al. 2013; Muralidharan et al. 2014; Chakrabarty et al. 2018; Shepherd et al. 2018a, b). In addition, a phase II clinical trial demonstrate that AT_2_R inhibition reduced neuropathic pain in individuals with post-herpetic neuralgia, supporting efficacy and safety for human treatment (Rice et al. 2014). Despite the growing interest, is still unclear whether AT_2_R plays any role in the development of pain and inflammation in gout. The purpose of the present study was to investigate the therapeutic potential of AT_2_R antagonism in alleviating the pain and inflammation of gout, by the use of an experimental mouse model.

## 2 Methods

### 2.1 Animals

All animal handling and experimental procedures were approved by the Ethics Committee in Animal Experimentation of the Federal University of Uberlândia (CEUA/UFU-080/16) or by the Animal Welfare Ethical Review Board (AWERB) of King's College London (for experiments in KCL). Adult male C57BL/6/J/UFU mice (20-25 g, bred in house) provided by UFU REBIR (UFU rodent animal breeding group), and C57BL/6N wild-type (WT) strain isogenic compared to Agtr2^tm1a^ mutant mice provided by KCL BSU (biological services unit), were used in the experiments. Agtr2^tm1a(EUCOMM)Wtsi^ (Agtr2^tm1a^) mutant mice were generated at Wellcome Trust Sanger Institute on a C57BL/6N genetic background (Skarnes et al. 2011; White et al. 2013). These mice carry a promoter-driven knockout-first allele, with a large cassette inserted in the intron before the targeted critical exon 3 which interferes with transcription leading to effective knockout of AT_2_R expression. Further details can be found at www.mousephenotype.org.

Animals were kept in a controlled-temperature environment in individual ventilated cages, with wood shaving bedding and nesting material, maintained at 22±1°C, with a 12 hours light/dark cycle and fed with rodent chow (Puro Lab 22 PB pelleted form, Global Diet 2018, Harlan, Lombardia for mice) and tap water *ad libitum*. Animals were allowed to acclimatize to their experimental room for 1 hour before experiments. Behavioral observations were performed in a blinded fashion by investigators and followed the Animal Research Reporting In vivo Experiments (ARRIVE) guidelines as well as (for experiments in KCL) in accordance with the Home Office (UK) regulations and the Animals (Scientific Procedures) Act 1986. Intra-articular injections were performed only in anesthetized mice (isoflurane 2%, 100% O_2_ 1L/min). The number of mice used in each experiment are presented in graph legends, and a total of 199 adult male mice were used for the study.

### 2.2 Reagents and Drugs

Unless otherwise indicated, all reagents were from Sigma (Sigma, St Louis, MO, USA) and dissolved using phosphate buffered saline (PBS) as vehicle. The AT_2_R antagonist, PD123319 ditrifluoroacetate, was purchased from TOCRIS Bioscience, USA (“1361” batch no: 3A/189254). MSU crystals were prepared according to Hoffmeister et al. (2011). Polarized light microscopic examination confirmed that the crystals were rod-shaped and varied in length (12 ± 2 μm). Crystals were aliquoted (100 μg) and kept stored for use only once, being discarded after use.

### 2.3 MSU-induced acute gout attack animal model and treatments

The acute gout attack animal model was induced by an intra articular (IA) injection of MSU crystals (10–100 μg/joint, typically 100 μg/joint, see Results) administered on into the tibio tarsal articulation (ankle joint) of the animals (Silva et al. 2016; Rossato et al. 2020).

The AT_2_R antagonist, PD123319, (10 pmol/joint) was co-administered by an intra articular injection with MSU crystals or Angiotensin II, or orally administrated (1 mg/kg) 30 min before MSU crystal IA injections (Muralidharan et al. 2014; Shepherd et al. 2018a). Angiotensin II was also administered alone (0.05–5 nmol/joint) by IA route (Shepherd et al. 2018a, with some modifications in relation to the route). After the injections the animals were analyzed for nociception and inflammation development at the time points 1, 2, 4, 6 and 24 hours.

### 2.4 Nociception evaluation

To evaluate behavioral nociception mice were placed in an acrylic cage individually (9 x 7 x 11 cm) with a wire grid floor, at least 1 hour before start of behavioral testing. When the animals had no exploratory movements, defecation and were not resting the evaluations began. Mechanical allodynia was measured in mice using von Frey hair filaments of increasing strength (0.008–1.4 g), applied in the center of the hind paw with a gentle stimulus following the “Up and Down” method as described by Chaplan et al. (1994). The weakest filament able to elicit a response was identified and the results were expressed as mechanical nociceptive threshold (Cunha et al. 2004). Spontaneous nociception was measured according to their behavior to support the weight of the body on the paw corresponding to the injected joint on a scale from 0 to 3 of spontaneous nociception (Coderre and Wall 1987; Silva et al. 2016). The cold nociceptive response were measured using a acetone cold stimulus (50 μl) that was sprinkled topically with the aid of a syringe to the center of the plantar surface of the hind paw (Caspani et al. 2009) with modifications. The online supplementary material provides detailed descriptions of the nociceptive procedures.

### 2.5 Inflammatory evaluation

As an inflammatory parameter we evaluated edema formation in the ankle joint 4 hours after MSU administration using a plethysmometer (Ugo Basile, Monvalle, Italy). The values were expressed in milliliters of water dislocated by the articulation and compared with the baseline measure or control groups.

To evaluate inflammatory neutrophil infiltration, we analyzed MPO activity and IL-1β levels. Only for this analysis we performed knee joint MSU (100 ug/joint) or angiotensin II (0.5 nmol/joint) injection, to reach the final volume necessary to the assays. Then, 4 h after MSU or angiotensin II injections, the injected joint (knee) synovial cavity was washed three times with 5 μL and the extract was diluted to a final volume of 50 μl of PBS to obtain the synovial lavage sample (Pinto et al. 2010; Rossato et al. 2020). Vehicle injected mice were used as a control. The samples were centrifuged at 800 g for 8 minutes at 4 °C, the pellet was collected and resuspended in 50 μL of PBS-EDTA for the MPO assay. The supernatant was collected and diluted in 20 μL of PBS-EDTA for IL-1β levels determination.

For MPO activity assessment, the resuspended pellet was homogenized in 80 mM NaPO_4_ buffer (pH 5.4) containing 0.5% hexadecyltrimethylammonium bromide (HTAB) and evaluated by colorimetric assay based on peroxidation of tetramethylbenzidine (TMB). The reaction was stopped by adding 4 M H_2_SO_4_ and determined by spectrophotometry (Spectra Max-250; Molecular Devices, Sunnyvale, CA, USA) at 450 nm. Results were presented as the number of neutrophils × 10^3^/mg of joint (Alves-Filho et al. 2010).

IL-1β was measured by ELISA following the manufacturer’s instructions (R&D Systems, Minneapolis, MN, USA). Results were expressed as picograms of cytokine per milligram of synovial fluid.

### 2.6 Nitric oxide (NO) concentration measurement

To evaluate the NO_2_ and NO_3_ articular tissue concentration, 4 h after PD123319, (10 pmol/joint) plus MSU, synovial lavage was obtained as previously described (Pinto et al. 2010). Samples were prepared as according to (Miranda et al. 2001; Rossato et al. 2020). Briefly 100 μl of standard nitrate solution (range 2 mM– 0.125mM) was serially diluted at 96-well plates, the collected samples were prepared with 100 μl of Griess reagent plus 40 μl of vanadium chloride (0.02mg/mL), then incubated for 1 hour at 37 °C. Measurements were made using a spectrophotometer with wavelength absorption (540 nm), and results were expressed in μM concentration.

### 2.7 RNA isolation and qPCR

For PCR analyzes, mouse tibio-tarsal articular whole joint samples were collected 4 hours after administration of MSU. The tissue was held in 500 μl of TRIzol reagent (Sigma-Aldrich, St. Louis, MO) and stored at -90 °C, until the day of the experiment, then the samples were homogenized with a Polytron Homogenizer (Thermo Scientific, USA). Quantity and purity of isolated RNA were checked by a NanoDrop spectrophotometer (Thermo Scientific, USA) with wavelength absorption ratio (260/280 nm) and 500 ng of RNA was transcribed into cDNA using reverse transcription reaction (Superscript II; Invitrogen Life Technologies). qPCR reactions have the final volume of 13 μl with 6.25 μl of PowerUp SYBR Green Master Mix (Applied Biosystems), 0.5 μl forward primer, 0.5 μl reverse primer, 4.75 μl Milli-Q water (Millipore Corporation) and 1 μl sample. Reactions were performed in 96-well plates compatibles with the Axygen Scientific Real-Time PCR System. Following initial denaturation, samples were cycled through denaturation (95 °C, 10 s), annealing (60 °C, 60 s) and extension (60 °C, 60 s) for 40 cycles, followed by melt curve analysis to ascertain specificity of amplification. Primers used (table 1).

### 2.8 Statistical Analyses

The number of animals needed in experiments was determined using the G. Power 3.1 software, statistical power greater than 7 was obtained. Kolmogorov-Smirnov normality test was used to determine whether the data values had normal distributions. Results were expressed as the mean±standard error of the mean (S.E.M.). Differences among 3 or more groups at one point were analyzed by one-way analysis of variance (ANOVA) followed by Newman-Keuls or Dunnett’s posttest. Differences among 3 or more groups at different times were analyzed by two-way ANOVA followed by Bonferroni’s posttest. Statistical analysis was performed using GraphPad Software 5.0 (GraphPad Software, San Diego, CA, USA). P values ≤ 0.05 were considered significant. To meet the ANOVA assumptions, the mechanical hyperalgesia data were log transformed prior to statistical analysis.

## 3 Results

### 3.1 AT_2_R activation is involved in nociception in MSU-triggered acute gout attack

**Figure 1:**
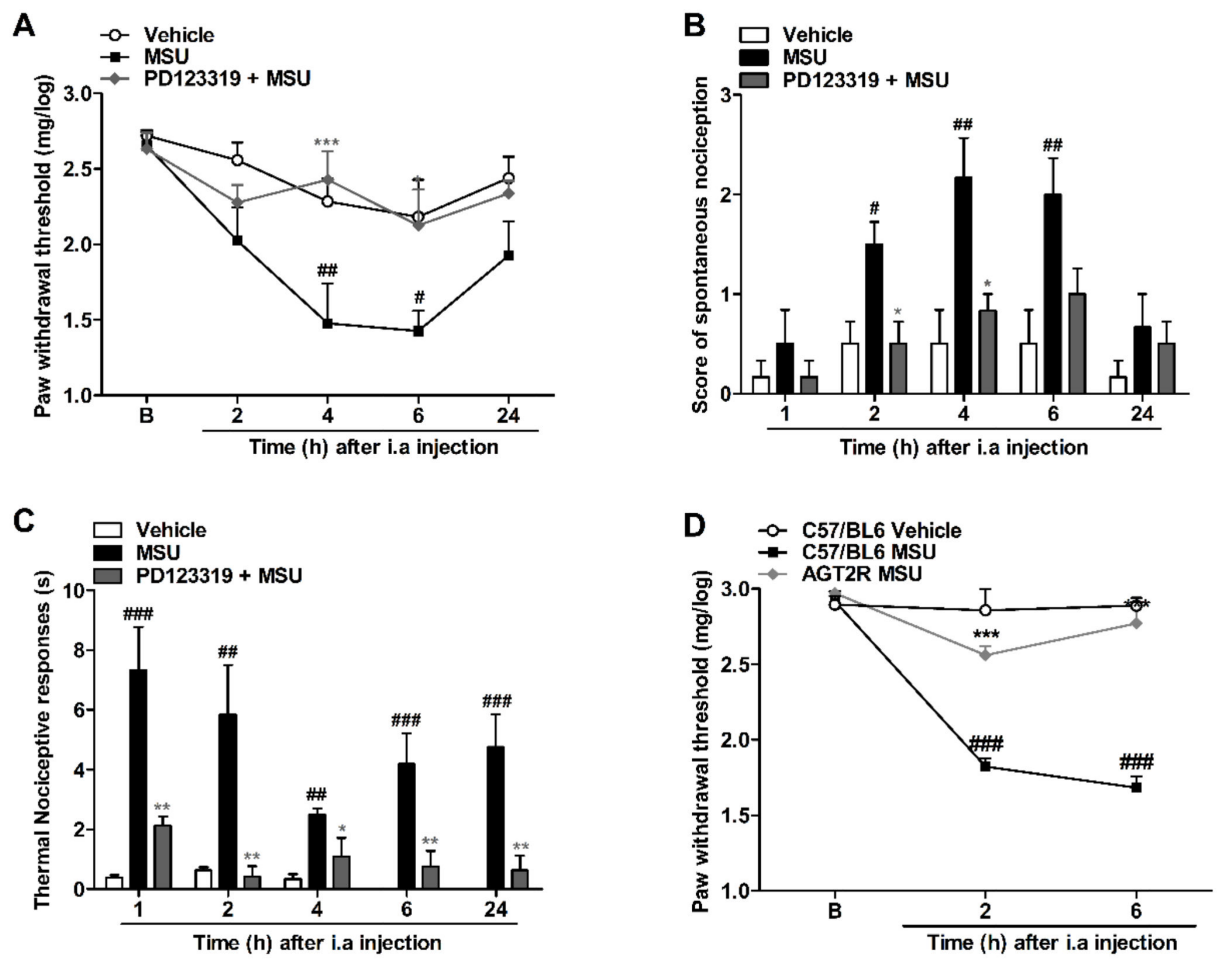
Prevention of MSU-induced nociceptive response mediated by treatment with angiotensin II type 2 receptor selective antagonist, PD123319, or Agtr2^tm1a^ mutant mice. (A and D) Mechanical allodynia, (B) spontaneous nociception, (C) thermal nociceptive responses. N = 6 mice per group. Each column represents the mean ± SEM. # P<0.05 and ## P<0.01 and ### P<0.001 represent significant differences compared to vehicle group. * P<0.05, ** P<0.01 and *** P<0.001 represent significant differences compared to MSU injected group. The statistical analysis was performed using two-way ANOVA followed by Bonferroni’s post-test in (A) and one-way ANOVA followed by Dunnet’s post-test in each interval (B), (C), (D).

**Figure 2:**
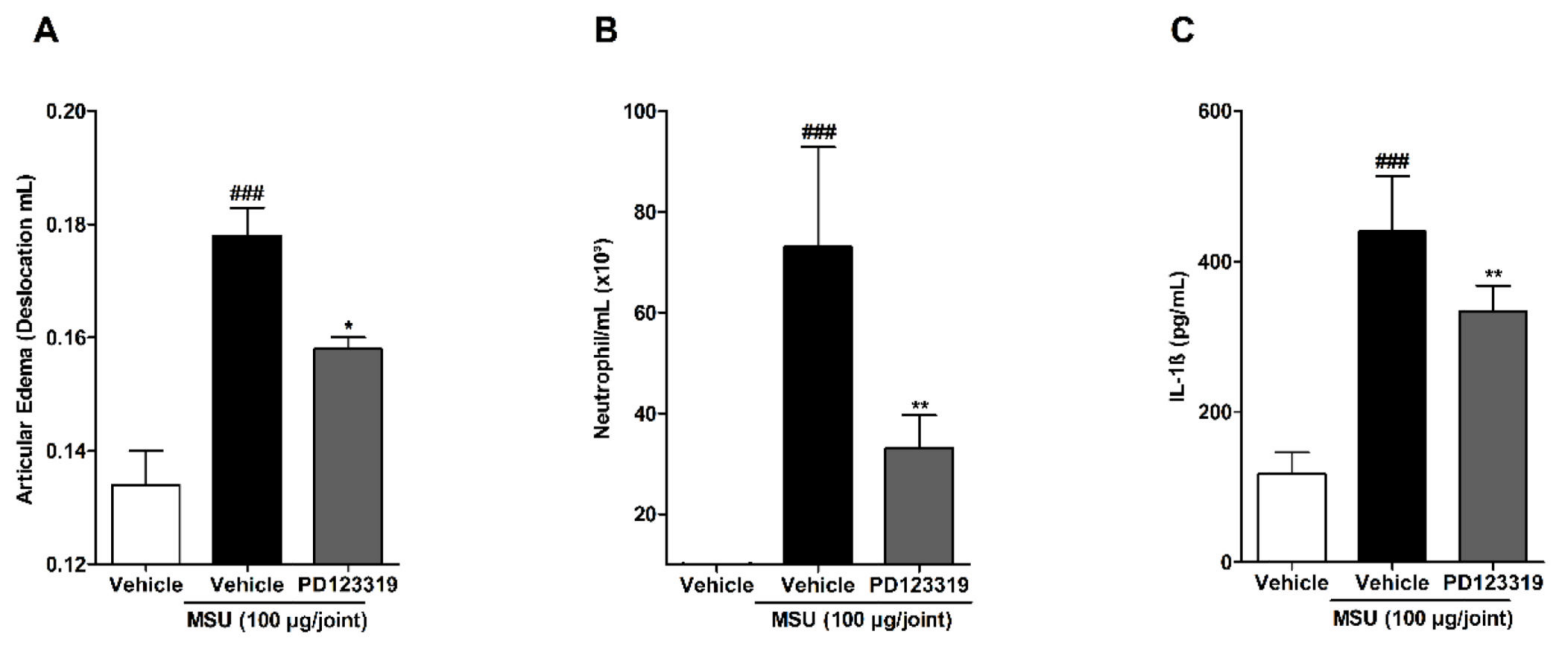
Prevention of MSU-induced inflammation mediated by treatment with angiotensin II type 2 receptor selective antagonist, PD123319. (A) Articular edema, (B) Myeloperoxidase activity and (C) IL-1β levels. N = 5 (A and B) and 10 (C) mice per group. Each column represents the mean ± SEM. ### P<0.001 represent significant differences compared to vehicle group. * P<0.05 and ** P<0.01 represent significant differences compared to MSU injected group. The statistical analysis was performed using one-way ANOVA followed by Dunnet’s post-test.

The MSU-induced acute gout attack model in mice was confirmed by the decreased paw withdrawal thresholds in response to mechanical stimulus, spontaneous and cold nociception development, when compared to the vehicle group (supplementary Fig. 1). The doses of 30 and 100 μg of MSU crystals evoked a significant nociceptive mechanical and spontaneous response, and the 100 μg dose was selected for following experiments (Rossato et al. 2020). Interestingly the intraarticular (IA) coadministration of the AT_2_R antagonist, PD123319 (10 pmol/joint) together with the MSU crystals (100 μg/joint), prevented mechanical allodynia at 4-6 h (Fig. 1A), spontaneous nociception from 2-4 h (Fig. 1B), and cold thermal nociceptive responses (Fig. 1C) from 1-24 h after IA injections. The same nociceptive parameters were analyzed for mice treated with the AT_2_R antagonist (PD123319, 1 mg/kg) given orally half hour before MSU IA injection. We found inhibition of both mechanical allodynia and spontaneous nociception 4-6 h after the injection (supplementary figure Fig. 2). Following this study, we routinely used IA rather than oral administration as it also allows the evaluation of AT_2_R involvement in acute gout employing lower quantities of the antagonist for the experiments.

To confirm the previous data, we induced the acute gout attack model in Agtr2^tm1a^ mutant mice, which are effectively deficient for the AT2 receptor. As expected, we noticed that IA injection of MSU (100 μg) in WT mice of the same strain (C57BL/6N) induced a significant reduction in the paw mechanical withdrawal threshold when compared to the PBS IA injection group (Fig. 1D). In agreement with the data obtained with PD123319 treatment, we observed that the Agtr2^tm1a^ mutant mice did not develop mechanical allodynia during MSU-triggered acute gout attack.

It has been demonstrated that AT_2_R can be expressed by macrophages (Shepherd et al. 2018b). Accordingly, we observed that peripheral macrophage depletion by administration of liposome-encapsulated clodronate leads to an antinociceptive response, as previously observed (supplementary Fig. 3A) (Rossato et al. 2020). The depletion of macrophages was confirmed by a viability test (supplementary Fig. 3B).

### 3.2 AT_2_R activation is involved in inflammation in MSU-triggered acute gout attack

An inflammatory process characterized by articular edema, neutrophil migration and increased IL-1β production was observed in the MSU-triggered acute gout attack model (Fig. 2A). The edema was prevented by local treatment with PD123319 (10 pmol/joint) (inhibition of 45.4±6.8 % of control, Fig. 2A), and the MSU-induced myeloperoxidase activity was also decreased in the PD123319-treated group in comparison with control group (54.6±4.9 %) (Fig. 2B). Moreover, PD123319 also prevented the IL-1β production (release) (32.7±18.4 %) (Fig. 2C).

### 3.3 Angiotensin II induced nociception is prevented by AT_2_R antagonism

**Figure 3:**
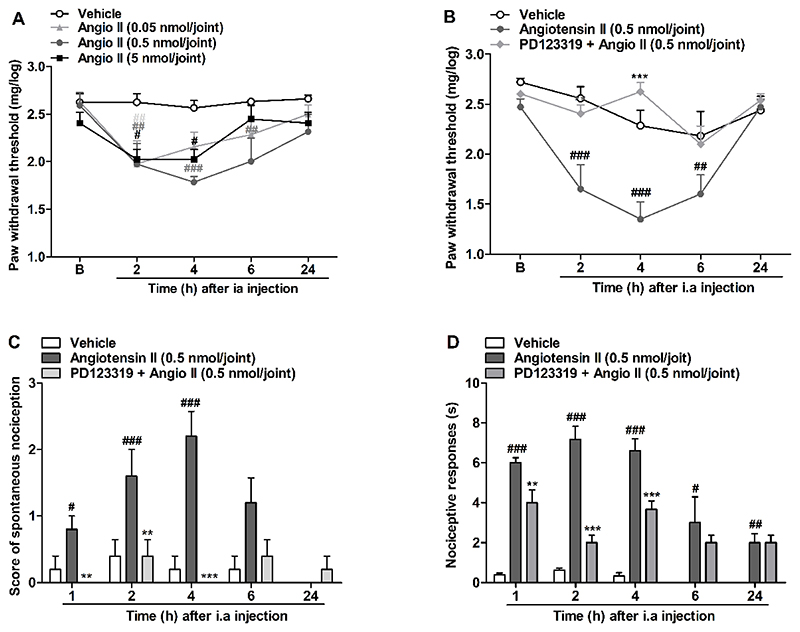
Articular nociceptive responses induced by angiotensin II and its prevention mediated by angiotensin II type 2 receptor selective antagonist, PD123319. (A and B) Mechanical allodynia, (C) spontaneous nociception and (D) cold thermal nociceptive responses. N = 6 mice per group. Angiotensin II (Angio II). Each column represents the mean ± SEM. # P<0.05 and ## P<0.01 and ### P<0.001 represent significant differences compared to vehicle group. * P<0.05, ** P<0.01 and *** P<0.001 represent significant differences compared to Angiotensin II injected group. The statistical analysis was performed using two-way ANOVA followed by Bonferroni’s test (A) and (B) and one-way ANOVA followed by Dunnet’s post-test in each interval (C) and (D).

To assess a specific action of AT_2_R on articulation, we treated the mice by IA injection with the AT_2_R agonist, angiotensin II, and evaluated the development of mechanical allodynia. We noticed that the agonist injection induced mechanical allodynia at all tested doses starting 2 h after the injection (Fig. 3A). The 0.5 nmol dose was chosen to be used for following experiments. AT_2_R antagonist, PD123319 (10 pmol/joint) completely prevented mechanical allodynia induced by angiotensin II (Fig. 3B). Angiotensin II was also able to induce spontaneous nociception and cold thermal nociceptive responses from 1-4 h, which was prevented by IA coadministration of the AT_2_R antagonist, PD123319 (10 pmol/joint) (Fig. 3C and D).

### 3.4 Angiotensin II induced inflammation is prevented by AT_2_R antagonism

**Figure 4:**
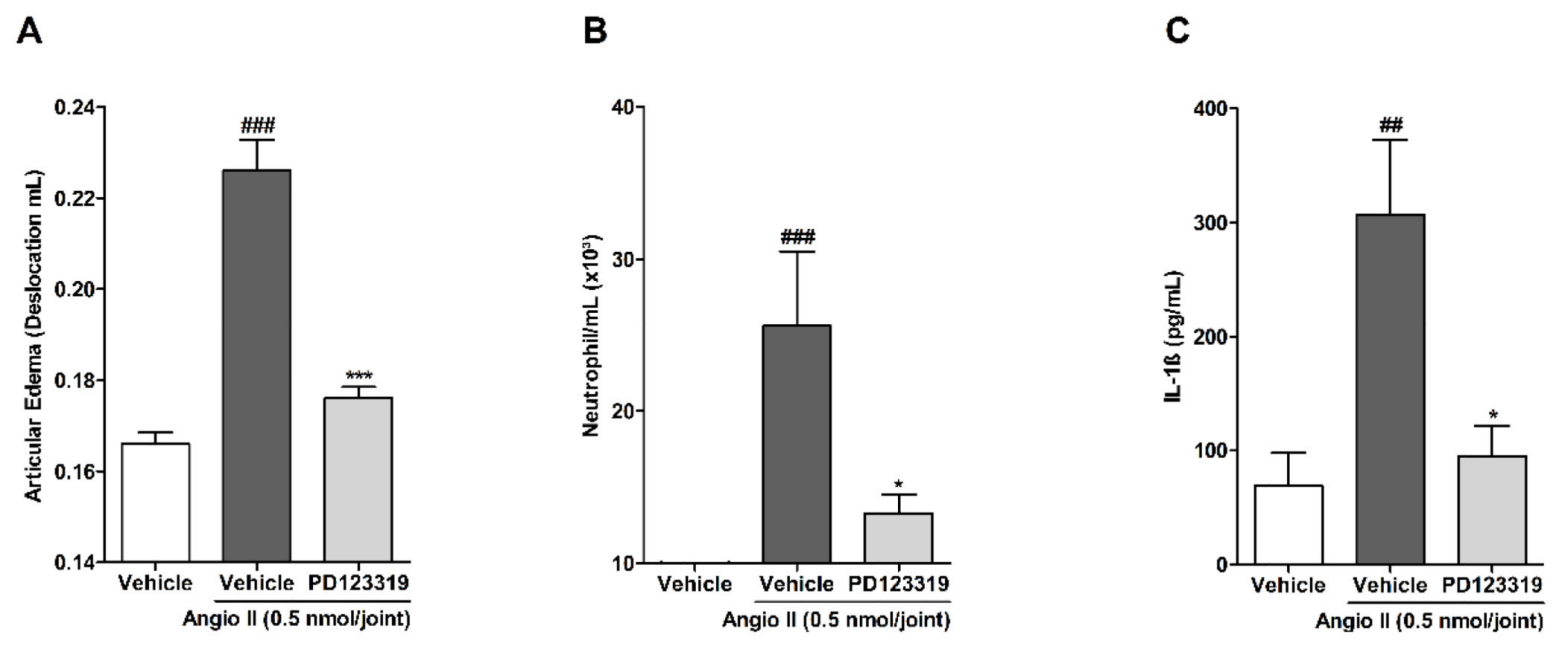
Prevention of angiotensin II inflammation mediated by treatment with angiotensin II type 2 receptor selective antagonist, PD123319. (A) Articular edema, (B) Myeloperoxidase activity and (C) IL-1β levels. N = 5 (A and B) and 6 (C) mice per group. Each column represents the mean ± SEM. ## P<0.01 and ### P<0.001 represent significant differences compared to vehicle group. * P<0.05 and *** P<0.001 represent significant differences compared to Angiotensin II injected group. The statistical analysis was performed using one-way ANOVA followed by Dunnet’s post-test.

To further explore the inflammatory role of AT_2_R on articulation, we treated mice with the AT_2_R antagonist plus angiotensin II, and evaluated edema, neutrophil migration and IL-1β production. The angiotensin II injection induced articular edema, neutrophil migration and increased IL-1β production additionally to the nociception previously observed. Interestingly, the coadministration of PD123319 plus angiotensin II reduced edema (83.33 % of reduction) (Fig 4A), myeloperoxidase activity (48.7±2.6 %) (Fig 4B) and IL-1β production (release) (89±26.4 %) (Fig. 4C).

### 3.5 AT_2_R antagonism prevented release of nitric oxides in MSU-induced acute gout model

**Figure 5:**
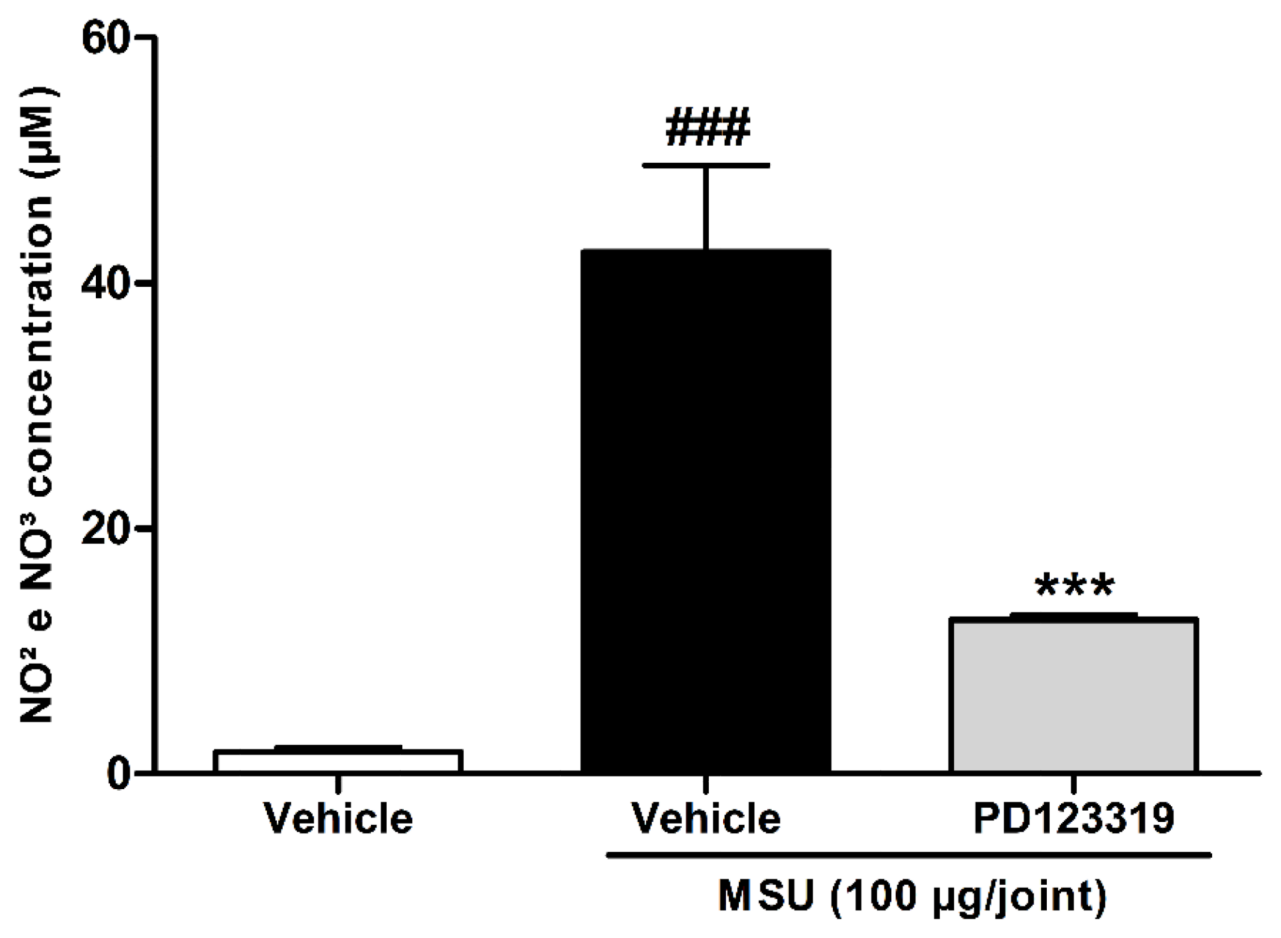
Prevention of NO levels in synovial fluid of mice submitted to acute gout attack mediated by treatment with angiotensin II type 2 receptor antagonist, PD123319. N = 6 mice per group. Each column represents the mean ± SEM. ### P<0.001 represent significant differences compared to vehicle group. *** P<0.001 represent significant differences compared to MSU injected group. The statistical analysis was performed using one-way ANOVA followed by Dunnet’s post-test.

Nitric oxides (NOX) are involved in rodent and human acute gout attacks (Carey et al. 2001; Dao et al. 2016; Gumanova et al. 2017; Rossato et al. 2020). In agreement, the NO_2_ and NO_3_ concentration in the articular synovial fluid of the MSU injected group were found to be significantly increased compared to the vehicle injected group (Fig. 5A). Co-administration of the AT_2_R antagonist, PD123319 (10 pmol/joint), significantly prevented the MSU-increased NO concentration (70±1 % of prevention) (Fig. 5A).

### 3.6 AT_1_R, AT_2_R, and ACE2 mRNA levels are altered in the ankle joint after MSU injection and AT_2_R antagonism

**Figure 6:**
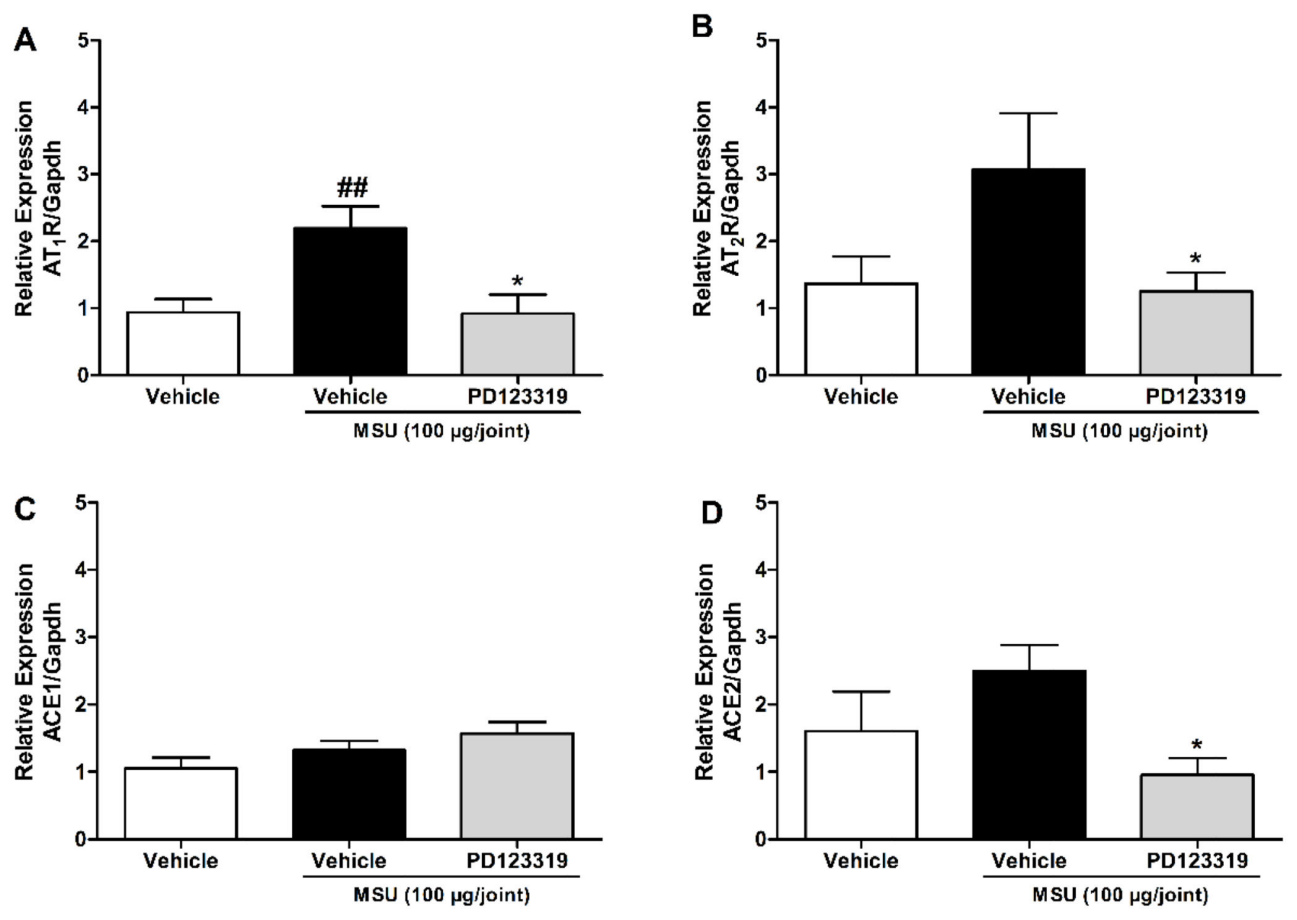
mRNA levels of AT_2_R, AT_1_R and ACE2 alterations in the acute gout attack model, and in response to angiotensin II type 2 receptor antagonist, PD123319. (A) AT_1_R mRNA levels, (B) AT_2_R mRNA levels, (C) ACE1 mRNA levels and (D) ACE2 RNA levels. N = 6-9 mice per group. Each column represents the mean ± SEM. ## P<0.01 represent significant differences compared to vehicle group. * P<0.05 represent significant differences compared to MSU injected group. The statistical analysis was performed using one-way ANOVA followed by Dunnet’s post-test.

We found an increase in AT_1_R mRNA levels in the acute gout attack model suggesting increased expression of the AT_1_R when MSU was intra-articular administered, which was decreased when AT_2_R was antagonized with PD123319 (Fig. 6A). Despite the slight altered mRNA levels of AT_2_R and ACE2, neither was statistically significant when comparing vehicle to the MSU group (Fig. 6B and D). However, when MSU plus PD123319-treated group was compared to MSU group, the mRNA levels of AT_2_R and ACE2 decreased significantly in both groups (Fig 6B and D). The results of the ACE1 qPCR shows no difference between the groups (Fig. 6C).

**Figure 7:**
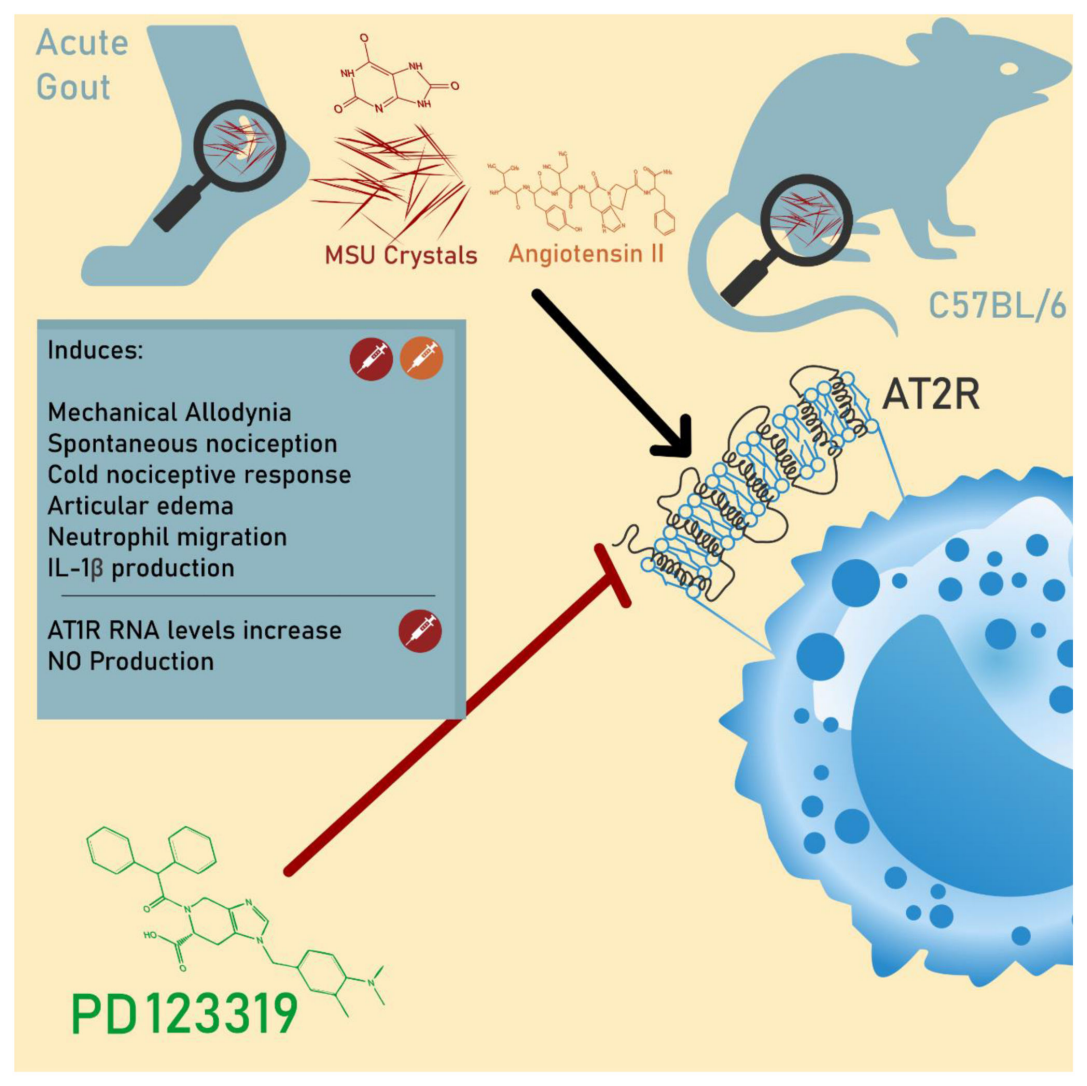
Possible mechanisms that account for the AT_2_R contribution for the development of acute gout attacks described in the study. AT_2_R = Angiotensin Receptor Type 2, IL-1β = Interleukin 1 beta, MSU = Monosodium Urate, NO = Nitric Oxide. CorelDRAW 2021 software were used to create artwork.

Collectively, all results described until here, indicate that AT_2_R involvement in gout includes NO and IL-1β release signaling as depicted in Figure 7.

## 4 Discussion

Gout is characterized by joint MSU crystal deposition, resulting in disabling and excruciating painful acute episodes (Dalbeth et al. 2019, 2021; Dehlin et al. 2020). Recent studies indicate that the angiotensin system is involved in pain sensitization, including in inflammatory conditions (Chakrabarty et al. 2018; Shepherd et al. 2018a, b), but this has not yet been shown for the pain of an acute gout attack. Here, we demonstrate that the antagonism of the AT_2_R prevented an acute gout attack in an animal model, alleviating pain and inflammation, and therefore that AT_2_R antagonists may contribute to a better management of gout.

Pain in gout is clinically described as disabling, characterized mainly by spontaneous pain and joint allodynia, and individuals affected by this condition have several problems in performing basic functions, such as walking. This strongly affects the patients’ quality of life, causing numerous public health, economic, and social problems (Busso and So 2010; Taylor et al. 2015; Dalbeth et al. 2019). In accordance, we confirmed pain development after MSU injections and, importantly, we demonstrated that AT_2_R antagonism has antinociceptive effects in an acute gout attack. Also, we verified for the first time that AT_2_R genetic deletion can prevent MSU inducing mechanical allodynia, suggesting an important role of the angiotensin system in the context of the arthritic pain of gout. AT_2_R pharmacological blockade has been described previously as a strategy to inhibit neuropathic, inflammatory and bone cancer pain in animal models (Smith et al. 2013, 2016; Muralidharan et al. 2014; Shepherd et al. 2018b), and in this study we extend the beneficial effects of AT_2_R inhibition to the pathology of gout.

Besides pain, we observed that the AT_2_R antagonism was also able to reduce articular edema, neutrophil infiltration and IL-1β release. MSU crystal injection reproduces in rodents the inflammatory characteristics observed in gout patients, such as redness, articular edema, neutrophil migration, as well as increased levels of IL-1β (Dalbeth et al. 2019). It is important to note that neutrophils are the main cells present in gout synovial fluid in humans and that IL-1β is the key cytokine driving the inflammatory process of an acute gout attack (Mitroulis et al. 2013; Dumusc and So 2015; So and Martinon 2017). These findings suggest that AT_2_R antagonism has the potential to treat acute gout attacks and other acute inflammatory conditions.

Although AT_2_R has recently been described to play a role in pain sensitization, there are few studies demonstrating the AT_2_R expression in articular tissues (Kawakami et al. 2012; Tsukamoto et al. 2013; Kawahata et al. 2015) and the recent literature suggests that angiotensin II does not directly influence sensory neuronal function (Shepherd et al. 2018a). Interestingly, after injecting angiotensin II into a naive mice ankle joint, we observed pain development, which was prevented by specific AT_2_R antagonism. Moreover, intraarticular angiotensin II administration also induced inflammatory parameters, that are also the clinical features of gout in humans, such as edema, neutrophil infiltration and IL-1β release, all of which were prevented by specific AT_2_R antagonism. These exciting findings from our investigation point out that the angiotensin system is an important player for the development of pain and inflammation in articular disease. In support of this proposal, it has been shown previously that MSU can increase articular ACE activity, and can also induce an increase in angiotensin II formation, which would be able to activate the AT_2_R in the articular microenvironment leading to an acute gout attack (Silva et al. 2016; Vargas Vargas et al. 2022).

Although the molecular mechanisms of these events remain to be elucidated, Shepherd et al. (2018a) suggested the existence of crosstalk between peripheral macrophages and sensory neurons, mediated by AT_2_R via TRPA1 redox signaling, as critical for peripheral pain sensitization. Macrophages are also present in the articular microenvironment and are involved in MSU-induced pain and inflammation (Martin et al. 2009; Rossato et al. 2020). Additionally, TRPA1 redox signaling has also been previously described as critical for an acute gout attack in mice (Trevisan et al. 2014). Altogether, these findings indicate that AT_2_R expression in macrophages may be related to articular pain and inflammation, such as observed in gout arthritis.

Besides TRPA1 involvement in gout, TRPV1 channels are also described as important for gout pain and inflammation (Hoffmeister et al. 2011; Rossato et al. 2020). More specifically, we have recently demonstrated that increased levels of nitric oxide, triggered by TLR4 expressed in phagocytic cells, results in TPRV1 activation and 1L-1β release during acute gout attack (Rossato et al. 2020). Nitric oxide and the enzyme responsible for its production, the inducible nitric oxide synthase, have been shown to be present in the synovial fluid of patients with gout and in MSU-stimulated cell culture (Chen et al. 2004). Interestingly, AT_2_R are upregulated by NO in endothelial cells, and AT_2_R activation leads to an increased synthesis and release of NO (Carey et al. 2001; Dao et al. 2016). In agreement, we find here that an MSU-induced gout attack is dependent on AT2 expression and NO release by macrophages, which may interact with TRP channels to cause IL-1β increase. These results point to AT_2_R as a new and important target to improve gout management.

Furthermore, we have observed that MSU increased articular AT_1_R gene expression, and the AT_2_R antagonism was able to reduce AT_1_R, AT_2_R and ACE2 gene expression, when compared to MSU-induced gout group. These observations confirm the complex regulation of the angiotensin system, where each receptor and enzyme can counteract to the expression/activity regulation of the others, as previously demonstrated (AbdAlla et al. 2001; Kostenis et al. 2005; Nemoto et al. 2014; Forte et al. 2016).

In summary, we have demonstrated an important role of AT_2_R in gout arthritic pain and inflammation, indicating that the involvement of AT_2_R in gout promotes the release of the pro-inflammatory factors NO and IL-1β, as depicted in Figure 7, and therefore may be a promising therapeutic target to improve the management of acute gout attacks.

## Supplementary Material

Figure S1

Figure S2

Figure S3

## Figures and Tables

**Table 1:** 

Name of primer	Primer sequence for 5’ 3’
AT_1_R-F	GGCCAGTGTTTTTCTTTTGAATTTAGCAC
AT_1_R-R	TGAACAATAGCCAGGTATCGATCAATGC
AT_2_R-F	CTGCTGGGATTGCCTTAATG
AT_2_R-R	CATCTTCAGGACTTGGTCAC
ACE-F	CACTATGGGTCCGAGTACAT
ACE-R	ATCATAGATGTTGGACCAGG
ACE2-F	GTGCACAAAGGTGACAATGG
ACE2-R	ATGCGGGGTCACAGTATGTT
GAPDH-F	GGGTGTGAACCACGAGAAAT
GAPDH-R	CCACAGTCTTCTGAGTGGCA

## Data Availability

The data that support the findings of this study are available on request from the corresponding author Silva C. R. The data are not publicly available due to the inexistence of free repositories where we can do that in a safe way.
